# Regulation of Drug Prescribing Information in Latin America and the Caribbean

**DOI:** 10.1007/s43441-022-00396-y

**Published:** 2022-04-05

**Authors:** Mariana Ramírez-Telles, Urimara Argotti-Rodríguez

**Affiliations:** 1 Regulatory Affairs – Medical Affairs Department, Roche Central America and the Caribbean, Heredia, Costa Rica; 2 International Regulatory Policies Department - Latin America, Productos Roche, S.A. de C.V., Mexico City, Mexico

**Keywords:** Prescription drugs, Drug regulations, Drug labeling, Insert, *Electronic labeling*, *ePI*

## Abstract

**Objective:**

To describe the status of drug regulations in Latin America and the Caribbean, in force as of May 2021, and assess through a comparative exercise the differences between the countries under scope on prescribing information of drugs for human use.

**Materials and Methods:**

A narrative review allowed the identification of the regulations concerning the prescribing information of drugs in 25 countries in Latin America and the Caribbean for the registration of prescription medications. On this basis, terms and concepts regarding this topic, the general provisions by the regulatory authorities for these products, applications for health registration and further amendments were identified for each country.

**Results:**

The Latin American and the Caribbean countries included, manage and regulate drug prescribing information differently in terms of concepts, information publishing, structure for product information, among other criteria. Few health authorities publish product information on their website. Additionally, the patient information leaflet is not requested for prescription drugs in most of the studied countries. There is no standardized structure for drug product information within the region**.**

**Conclusions:**

A poor level of harmonization among the regulations from these countries regarding the content and management (e.g. if physical package insert is required or not, if it is subject to notification or approval) of the prescribing information of human use drugs became evident. Also, there is a visible lack of standardization of concepts for referring to a specific document (e.g., package insert for healthcare professionals, patient information leaflet and technical information for the drug product) and in the content itself.

## Introduction

The information concerning prescription drugs is highly relevant for their rational use. According to the European Medicines Agency (EMA), the information on a pharmaceutical product corresponds to the approved and official documents on drugs for health professionals and patients, including the Summary of Product Characteristics (SmPC) (simplified in the study as product information/monograph), the package insert, and the labeling [1]. The product information/monograph and Patient Information Leaflet (PIL) are the focus for the assessment in this paper. This paper does not cover the information or data present in drugs' primary, secondary, or tertiary packaging material. Prescription drugs are hereby understood as those that are prescribed to a patient for a specific use by a qualified professional using a written instruction [2–4].

Thus, the package insert for patients and the information for prescribers have the purpose of offering key information for the proper adequate use of the pharmaceutical product and facilitate its understanding [5]. Both are acknowledged as important tools for health education, however, difficulties in their legibility and understanding have been reported in several publications [6–9]. This problem results from variables such as health literacy, since health information should reach the general population through a simple and concise language [9]. Therefore, it becomes important to emphasize the role of adequate information for patients' safety, whose contents include plain language for easy interpretation, even for users with a low general educational level [10].

Studies have acknowledged prescribing information as a reliable and useful tool for the everyday practice of pharmacists and other health professionals [11]. The format and contents of drug prescribing information are determined in legal terms and evolve over time [12]. A gap has been identified between the current content in the product information and the one that could be more useful for patients [13]. Covering this need requires flexibility to modify the volume of information and data included according to the actual needs [13]. Moreover, literature has shown that regulations for labeling are inconsistent across this region [14]. This previous conclusion has also been registered for countries such as the United States, Canada and the United Kingdom and the authors from this study confirmed that safety information should be aligned across elements such as its length and content [15].

There are no current studies on the assessment of labeling regulations in this region. Most of the available data comes from the United States, Canada or countries from Europe. Therefore, this investigation aims to highlight insights intended to generate evidence that could serve for promoting further decision-making in health ecosystems and encouraging convergence between the different national regulatory systems for elements such as content, format, readability, regulation, among others. The harmonization of requirements and standardization of format and processes will contribute in adopting global structured product labeling standards for labeling information [16], improving the data exchange with other relevant developments in the Digital Health ecosystem such as electronic medical records, e-prescriptions and pharmacovigilance [17], and fullfiling gaps from the user experience perspective. Thus, the objective of this paper is to describe the status of regulations for the prescribing information of drugs for human use in Latin America and the Caribbean in force as of May 2021. Likewise, the secondary objectives are identifying the elements that might contribute to labeling creation and the implementation of electronic media for their dissemination across the region.

## Methods

A search for regulatory frameworks in force as of May 2021 concerning the registration of prescription drugs in Latin America and the Caribbean was conducted. The regulations included were those describing elements of the information targeted at health professionals (e.g.,product information/monograph) and for patients (package insert or leaflet, according to each document). Those documents that did not contain this matter were excluded from the study and were not part of the scope of this project.

Each regulatory authority's current electronic site was checked to determine if the information of interest for this research was published there, allowing the identification of the terms and definitions related to product information and package inserts that are aimed for healthcare professionals and patients and which are found in the regulation from each country. The search was conducted in 25 countries from Latin America: (Argentina, Bolivia, Brazil, Chile, Colombia, Costa Rica, Cuba, Ecuador, El Salvador, Guatemala, Honduras, Mexico, Nicaragua, Panama, Paraguay, Peru, Dominican Republic, Uruguay and Venezuela) and Caribbean (Aruba, Curacao, Guyana, Jamaica, Sint Maarten, Trinidad & Tobago), also referred as the Caribbean Community (CARICOM).

Subsequently, a narrative synthesis of the results and summary tables categorizing the identified information were developed. A comparison of the regulations was made to compare these parameters: each country’s requirements to identify ifinformation for healthcare professionals or patients is required), the National Regulatory Authority—NRA requested format (in paper or digital), and the general process from the regulatory standpoint for the management of updates throughout the product life cycle, according to the type of modification (safety or efficacy). Furthermore, there was a search to recognize if the product information is published by the NRA on their website to allow public access.

## Results

The regulations [18–60] used for the narrative synthesis are listed by country in Table 1.

**Table 1:** Regulations in Force by May 2021 Concerning the Labeling of Prescription Drugs for Human Use in Latin America and the Caribbean [18–60]

Country	Regulatory authority (NRA)	Reference regulation	Website
Argentina [18–20]	Argentina’s National Administration of Drugs, Food, and Medical Technology (ANMAT)	Provision 5904/1996: “Definitions and general guidelines about the way information shall be presented in inserts of medical specialties wholesale condition is under the three categories of prescription” Executive Order Nº 150/1992 Norms for the registration, creation, fractioning, prescription, sale, marketing, exportation, and importation of medical products. Application field. General Provisions Circular Letter Nº 004/2013: Insert: Information for Patients	https://www.argentina.gob.ar/anmat
Aruba	Drug Registration Board—Aruba	There is no specific regulation	No specific website identified https://www.government.aw/
Bolivia [21–23]	Federal Agency of Drugs and Health Technologies (AGEMED)	Regulation for the Medication Law Supreme Executive Decree. 25235 Sanitary Registration Manual Ethical Norms for the Promotion of Medications	https://www.agemed.gob.bo/
Brazil [24]	Sanitary Surveillance National Agency (ANVISA)	Resolution RDC N° 47, September 08, 2009	https://www.gov.br/anvisa/pt-br
Chile [25–27]	Public Health Institute (ISP)	Supreme Decreet N° 3/2010 Instructions for the contents of the information leaflet for professionals Instructions for the contents and format of the information leaflet for patients	https://www.ispch.cl/
Colombia [28, 29]	National Institute for Drug and Food Surveillance (INVIMA)	Executive Order 677, 1995 Guideline for the presentation of amendments to the sanitary registration for the Medicinal and Biological Products Directorate	https://www.invima.gov.co/
Costa Rica [30–33]	Directorate of Regulation of Products of Health Interest, Ministry of Health (DRPIS)	Central American Technical Regulation Nº11.03.59:11, Pharmaceutical Products, Medications for Human Use Requirements. Reg. Sanitary Central American Technical Regulation Nº RTCA 11.01.02:04 Labeling of Pharmaceutical Products for Human Use Technical Regulation: RTCR 440: 2010. Regulation for the Inscription and Control of Biological Medications Executive Order N° 39433 Acknowledgment of the evaluation and approval of final reports of clinical and non-clinical trials by the regulatory authorities of reference as evidence for the Sanitary Registration of Medications	https://www.ministeriodesalud.go.cr/
Cuba [34, 35]	Center for the State Control of Medication Quality (CECMED)	Regulation M 83-15. Requirements for the Sanitary Registration of Biological Products for Human Use (Annex 3B) Regulation N.° 61-2012: Requirements for the Sanitary Registration of Medicinal Products for Human Use (Annex 3 M)	https://www.cecmed.cu/
Curacao [36]	Inspectorate of Public Health—Bureau of Pharmaceutical Affairs	Guidelines for new drug registration	No specific website identified https://gobiernu.cw/nl/ministries/gezondheid-milieu-natuur/sector-gezondheid/
Ecuador [37, 38]	National Agency for Sanitary Regulation, Control and Supervision (ARCSA)	Ministerial Agreement 385: Regulation for obtaining the Sanitary Registration for Biological Medications Ministerial Agreement 586: Regulation for the Sanitary Registration of Medications in General	https://www.controlsanitario.gob.ec/
El Salvador [39]	National Bureau of Medications (DNM)	Central American Technical Regulation Nº11.03.59:11, Pharmaceutical Products, Medications for Human Use Requirements. Reg. Sanitary Central American Technical Regulation Nº RTCA 11.01.02:04 Labeling of Pharmaceutical Products for Human Use Guideline for the Registration of Biological and Biotechnological Medications	https://www.medicamentos.gob.sv/index.php/es/
Guatemala	Department for the Regulation and Control of Pharmaceutical Products and the like—Ministry of Public Health and Social Welfare	Central American Technical Regulation Nº11.03.59:11, Pharmaceutical Products, Medications for Human Use Requirements. Reg. Sanitary Central American Technical Regulation Nº RTCA 11.01.02:04 Labeling of Pharmaceutical Products for Human Use	https://www.mspas.gob.gt/
Guyana [40]	Food and Drug Department—Ministry of Health	Drug Registration Scheme	https://www.health.gov.gy/
Honduras	Health Regulation Agency (ARSA)	Central American Technical Regulation Nº11.03.59:11, Pharmaceutical Products, Medications for Human Use Requirements. Reg. Sanitary Central American Technical Regulation Nº RTCA 11.01.02:04 Labeling of Pharmaceutical Products for Human Use	https://www.arsa.gob.hn/
Jamaica [41]	Ministry of Health and Wellness	List of Requirements for the Registration of a New Drug	https://www.moh.gov.jm/
México [42–47]	Federal Commission for the Protection against Sanitary Risk (COFEPRIS)	Regulation of the General Health Law for Advertising Health Supplies Advertising, Health Supplies Regulation Official Mexican Standard NOM-059, Good Manufacturing Practices for Medicinal Products Official Mexican Standard NOM-072-SSA1-2012, Labeling of medicinal products and herbal remedies Official Mexican Standard NOM-220, Pharmacovigilance Installation, and Operation	https://www.gob.mx/cofepris
Nicaragua [48]	Sanitary Regulation Directorate-General—Ministry of Health^a^	Central American Technical Regulation Nº11.03.59:11, Pharmaceutical Products, Medications for Human Use Requirements. Reg. Sanitary Central American Technical Regulation Nº RTCA 11.01.02:04 Labeling of Pharmaceutical Products for Human Use NTON 19 013-20 Medications for Human Use. Products of Biologic Origin and Biosimilars, Registration Requirements	http://www.minsa.gob.ni/
Panamá [49]	National Directorate of Pharmaceutical Products and Drugs—Ministry of Health	Executive Order N° 95. Tuesday, May 14, 2019 Central American Technical Regulation Nº RTCA 11.01.02:04 Labeling of Pharmaceutical Products for Human Use	https://www.minsa.gob.pa/direccion/direccion-nacional-de-farmacia-y-drogas
Paraguay [50–52]	Sanitary Surveillance National Directorate (DINAVISA)	Resolution DNVS N° 84. Guideline for the assessment of applications of registration for pharmaceutical specialties of synthetic or semi-synthetic origin Order 6611/16 Requirements for the Registration of Biological Medications Act 1119/97 on Health Products and Others	https://www.mspbs.gov.py/dnvs
Perú [53]	Directorate-General of Medications, Supplies, and Drugs (DIGEMID)	Supreme Order N° 016-2011-SA: Regulation for the Registration, Control, and Surveillance of Pharmaceutical Products, Medical Devices, and Sanitary Products	https://www.digemid.minsa.gob.pe/
Dominican Republic [54, 55]	General Directorate of Drugs, Food, and Sanitary Products (DIGEMAPS)	Order N.° 246-06 Regulation on the manufacturing, creation, quality control, supply, circulation, distribution, marketing, information, advertising, import, storage, disposition, assessment, registration, and donation of medications Technical regulation for the sanitary registration of innovative and non-innovative biotechnological medicines for human use in the Dominican Republic	https://digemaps.msp.gob.do/menu/
Sint Maarten	Pharmaceutical Registration Committee of Sint Maarten	There is no specific regulation	No specific website identified http://www.sintmaartengov.org/Pages/default.aspx
Trinidad & Tobago [56]	Chemistry, Food and Drugs Division—Ministry of Health	Summary of requirements for new drug submission	https://health.gov.tt/services/chemistry-food-and-drugs-division
Uruguay [57–59]	Sanitary Evaluation Division—Ministry of Public Health	Order N° 18/020 Regulation for the registration, production, export, import, and marketing of Medicinal Products for Human Use Order 38/015 Registration of Biotechnological Medications Order 18/989 Information and advertising of Medications	https://www.gub.uy/ministerio-salud-publica/home
Venezuela [60]	National Hygiene Institute Rafael Rangel—INHRR	Norms from the Review Board for Pharmaceutical Products: General Norm for Inserts	http://www.inhrr.gob.ve/

^a^Pursuant to Act N.° 1068, approved on March 18, 2021 (published in La Gaceta, Official Gazette N° 58, dated March 24, 2021), the National Authority for Sanitary Regulation was created [75]

Table 2 describes the definitions related to the information on prescription drugs according to the regulations listed above in force by May 2021. We acknowledge that there are several regulations currently under review in countries such as Mexico. There is a wide variety of terms and definitions found in the regulations. At first glance, there is an evidentlack of standardization in this region given the diversity of concepts that are employed to refer to the drug product information (product information, monograph, information for patients, Summary of Product Characteristics, leaflet, package insert and others that can be retrieved in Table 2).

**Table 2:** Structure and Used Terms for the Information on Prescription Drugs for Human Use in Latin America and the Caribbean [18–60]

Country	Used terms and definitions	Is there a defined structure for the regulation of these requirements?
Argentina	The regulations lack explicit definitions. They mention an *insert for prescribing (product information/monograph) and “Information for the patient'',* the latter as an insert in lay language for comprehension by the general population	Yes
Bolivia	There are no specific definitions. The following two concepts are distinguished: *Scientific data (product information/monograph) and information for the patients and general public* (for inserts, which should contain information in simple language)	No, but it defines the minimal sections to be included
Brazil	*Package insert*: a legal sanitary document containing the technical and scientific information for the rational use of medications *Insert in a special format*: an insert targeted at individuals with a visual deficit in the adequate format for their needs *Insert for patients*: an insert intended for patients, approved by ANVISA, with a summarized content, in adequate language, and easy to understand *Insert for health professionals*: an insert targeted at health professionals, approved by ANVISA, with detailed technical content *Electronic insert*: ANVISA database, available on their website, containing the most recent approved versions of inserts for medications or other documents	Yes
CARICOM: Aruba, Guyana, Sint Maarten, Trinidad and Tobago	There is no information available	It is not clearly established
CARICOM: Curaçao, Jamaica	There is no information available	No, but it defines the minimal sections to be included
Central America: Costa Rica, El Salvador, Guatemala, Honduras and Nicaragua^a^	(*Product information/monograph*:) “*Product monograph***:** technical-scientific description of the safety profile and efficacy of a medication or pharmaceutical product “*Insert, leaflet or directions*: technical-scientific information included in the finished drug product, which must contain the necessary data for the safe and effective use of the contained medication.”	No, but it defines the minimal sections to be included
Chile	“*Monograph*: A document containing the technical, pharmaceutical, and scientific description of a product’s characteristics and properties *Information for health professionals*: An array of procedures and activities targeted at professionals legally enabled to prescribe or distribute pharmaceutical products, to show them the products this regulation refers to, in accordance with the authorization in the corresponding sanitary registration *Information leaflet for professionals*: A document containing at least the characteristics of the pharmaceutical specialty determined by the authority based on the scientific nature and information available for a pharmaceutical product, with the purpose to inform legally enabled professionals to prescribe and distribute pharmaceutical products *Information leaflet for patients*: A document intended to inform patients about a pharmaceutical specialty”	Yes
Colombia	In the requirements for sanitary registration, it is described as: *Pharmacological Information Summary.* It is also described as: *Insert for patients* and *Prescribing Information* (IPP)	No, but it defines the minimal sections to be included
Cuba	*Summary of Product Characteristics (SmPC)*: it is the information about a biological product created and approved by CECMED and targeted essentially at health professionals so that it can be prescribed, distributed, and used in a rational, safe and effective way *Information for Patients:* Information to be offered to patients, distributed on the primary and secondary packaging and insert with which the product is marketed*.”*	No, but it defines the minimal sections to be included
Ecuador	*Summary of Product Characteristics (SmPC) or Pharmacological data sheet* *Insert targeted at users and health professionals* *“Insert:* The informative leaflet for users included as part of the medication packaging.”	No, but it defines the minimal sections to be included
Mexico	"**Insert (Instructive or insert)**, the information that, in written or graphic format, explains the use or any other information regarding the rational use of the medication or herbal remedy, except for advertising, to users"	No, but it defines the minimal sections to be included
Panama	The definition of leaflet or insert is the one contained in Central American Technical Regulation 11.01.02.04—Labeling of Pharmaceutical Products for Human Use. Order 95, dated May 14, 2019, has no additional definitions	No, but it defines the minimal sections to be included
Paraguay	**“***Monograph***:** Technical and scientific description of a medical product, presenting information on its chemical nature, pharmacological activity, instructions for its adequate use, therapeutic usefulness, and dosing regimen *Insert***:** Printed information attached to the medication separately, presenting details on its qualitative and quantitative composition, identification, indications, adverse reactions, contraindications, and interactions. It is considered part of the labeling.”	No, but it defines the minimal sections to be included
Peru	“*Data sheet*: Technical and scientific information targeted at health professionals and approved by the National Authority form Medications, containing necessary, important and balanced information allowing adequate use and minimizing the risks associated with the use of the pharmaceutical product. *Insert***:** Written information targeted at the patient or user and accompanying the pharmaceutical product or medical device.”	Yes
Dominican Republic	*“Informative sheet for the pharmaceutical specialty professional*: A document summarizing the essential information on the pharmaceutical specialty, to share it with health professionals. The holder or owner of the registration authorization must share it with Medical professionals when registering it *Medication Data Sheet*: The document summarizing the essential information about the medication provided by the holder during the registration process, given the process of schematic information about the medication before the Directorate-General of Drugs and Pharmacies *Insert or leaflet*: The written literature targeted at consumers, customary within the packaging material for a pharmaceutical specialty, which is mandatory.”	No, but it defines the minimal sections to be included
Uruguay	There is no information available	No, but it defines the minimal sections to be included
Venezuela	There is no information available	Yes

^a^In Technical Norm NTON 19.013-20 on Products origin and Biosimilars, Registration Requirements, the monograph is also called Summary of Product Characteristics

Likewise, only 20% of the 25 countries (Argentina, Brazil, Chile, Peru, and Venezuela) have a clearly defined structure to be followed in terms of the sections' order, format, and content to be considered. The Caribbean countries lack explicit provisions concerning this topic in their valid norms and regulations. The rest of the countries in the study contain a list of the minimal sections to be included in the contents to be approved. However, the Summary of Product Characteristics (SmPC) published in Cuba’s CECMED web page comprises a standardized structure for all products.

Table 3 describes general aspects associated with the regulation of the information for prescription drugs in Latin American and Caribbean countries within the scope of this study. Twelve percent of the countries (3 out of 25) require adopting the contents approved by a reference authority. Concerning the presentation of inserts, 72% (18 out of 25) need an insert in physical format as part of the finished product, while 28% have not established this clearly. About 32% of the countries require pharmaceutical products to include a package insert targeted at patients and another targeted at health professionals. In addition, 8% of them just ask for the material targeted at patients and 60% require the product information only for health staff. Of note, all of the countries establish the approval of updates on the efficacy of medications as a requirement. Concerning procedures related to safety amendments, only Bolivia and Venezuela (8%) require submissions in the form of notifications, and the rest of the countries ask for approval, except for the Caribbean countries without a clear pathway, due to their lack of a specific regulation. Finally, the review of the authorities' websites listed in Table 1 showed that only 24% of the regulatory authorities in the region include the publication of the approved information for prescription drugs for human use.

**Table 3:** Comparative Table of the Management of Prescribing Information of Drugs for Human Use in Latin America and the Caribbean [18–60]

Country	Appearance						
	Must the information approved by a reference regulatory authority be adopted?	Is the prescribing information for health professionals (data sheet) required?	Is prescribing information for patients required?	Is the insert or leaflet described as a physical material?	Is approval for amendments related to the efficacy of medications required?	Is approval for amendments related to the safety of medications required?	Is the prescribing information published on the website of the regulatory authority?
Argentina	Yes	Yes	Yes	Physical material	Yes	Yes	Yes
Aruba	No	Yes	No	It is not clearly established	Yes	There is no formal regulation	No
Bolivia	No	Yes	Yes	Physical material	Yes	Only notification	No
Brazil	No	Yes	Yes	Physical material	Yes	It depends on the kind of amendment	Yes
Chile	No	Yes	Yes	Physical material	Yes	Yes	Yes
Colombia	No	Yes	No	Physical material	Yes	Yes	No
Costa Rica	No^a^	Yes	No	Physical material	Yes^b^	Only notification^c^	No
Cuba	No	Yes	No	It is not clearly established	Yes	Yes	Yes
Curacao	No	Yes	No	It is not clearly established	Yes	There is no formal regulation	No
Dominican Republic	No	Yes	Yes	Physical material	Yes	Yes	No
Ecuador	No	Yes	Yes	Physical material	Yes	Yes	No
El Salvador	No	Yes	No	Physical material	Yes^b^	Only notification^c^	Yes
Guatemala	No	Yes	No	Physical material	Yes^b^	Only notification^c^	No
Guyana	No	Yes	No	It is not clearly established	Yes	Only notification	No
Honduras	No	Yes	No	Physical material	Yes^b^	Only notification^c^	No
Jamaica	No	Yes	No	It is not clearly established	Yes	There is no formal regulation	No
Mexico	No	Yes	Yes	Physical material	Yes	Yes	No
Nicaragua	No	Yes	No	Physical material	Yes^b^	Only notification^c^	No
Panama	Yes	Yes	No	Physical material	Yes	Yes	No
Paraguay	No	No	Yes	Physical material	Yes	Yes	No
Peru	Yes	Yes	Yes	Physical material	Yes	Yes	Yes
Sint Maarten	No	Yes	No	It is not clearly established	Yes	There is no formal regulation	No
Trinidad & Tobago	No	Yes	No	It is not clearly established	Yes	There is no formal regulation	No
Uruguay	No	Yes	No	Physical material	Yes	Yes	No
Venezuela	No	No	Yes	Physical material	Yes	Only notification	Yes^d^

^a^Executive Order N° 39433 Acknowledgment of the evaluation and approval of final reports of clinical and non-clinical trials by the regulatory authorities of reference as evidence for the Drug Marketing Authorization

^b^It is considered a part of the heading established by the Central American Technical Regulation N° 11.03.59:11 as: Amendment A4 for Changes in the monograph and insert or A20 for the Extension of Therapeutic Indications

^c^It is considered a part of the heading established by the Central American Technical Regulation N° 11.03.59:11 as: Amendment B4 for Changes in the product's safety information

^d^In Venezuela, the web page of the National Hygiene Institute “Rafael Rangel” presents the pharmacological sheets of approved active ingredients

## Discussion

### General Provisions Identified in the Regulation

In regulatory terms, there are two kinds of documents that present information on the use of prescription drugs, according to the population they address: patients or health professionals. The terminology used to designate them is diverse, as shown in Table 2.

The package insert for patients seeks to orient the administration and the benefit/risk profile of a prescribed medication [61]. However, this review’s results show that, in the CARICOM and Central American countries, the existence of such material is not acknowledged. For Central America, the package insert [31] is only required for including precautions, contraindications, and warnings when they are absent from the secondary packaging label of a liquid dosage form and only the product information (monograph) is mandatory [30, 49, 50]. In Panamá [49], the insert is mandatory for the products listed as reference and their bioequivalents. In Bolivia [22], the insert is required when the minimal information (minimum sections outlined by the regulation in force) required cannot be found in the primary or secondary packaging.

One of the essential elements for creating an effective and efficient insert for patients is an appropriate language and length to improve the likelihood of its understanding and widespread reach [62]. In this regard, a contributing factor is health literacy, defined as the degree to which people can get, process, and communicate health-related information that is important for informed decision-making [63]. Argentina, Brazil, Bolivia, Chile, Colombia, and Cuba are some of the countries that consider the importance of a lay language and accessible documents to adapt the information designed for patients as a part of their regulatory framework. This raises the importance of promoting health literacy in the discussions for other countries so that they are able to include this requirement for improving the understanding of the information for patients and caregivers.

The Spanish Agency for Medicine and Health Products (AEMPS) [64] conceives in a general way the Summary of Product Characteristics: “It is the document intended for health professionals containing the indications, posology, recommendations for use, warnings and contraindications, adverse reactions, pharmacodynamic and pharmacokinetic properties, warnings for use in special populations, pregnancy, breastfeeding, and other relevant information.” Concerning the product information, as the information addressed to health professionals, there is no regional consensus about the term used for this document. The following different terms were identified: leaflet for prescribing [19], scientific data sheet [22, 23], insert for health professionals [24], monograph [30, 49, 51], professional information leaflet [26], prescribing information [28, 29], Summary of Product Characteristics [34, 35, 37, 38], data sheet [53] and information sheet.

Another relevant finding is that the prescribing information for drugs in Brazil (according to the type of pharmaceutical products), Mexico, and Uruguay can only be addressed to health professionals [24, 42–47, 57–59]. In Peru (the online version is targeted at health professionals), Argentina, Brazil (depending on the pharmaceutical product), Cuba, Peru, and the Dominican Republic establish that the insert is for patients or the general public.

The general population is increasingly aware of their health and wishes to receive further information on their medications [65]. A positive impact on the level of knowledge among patients due to the use of the insert for patients has been reported [66]. Similarly, the product information aids the management tasks handled by healthcare professionals [67]. Regulations from Argentina, Brazil, Chile, Dominican Republic, Ecuador, Mexico, Peru, and Nicaragua (in the norm for biotechnological drugs) consider documents for patients and healthcare professionals, while Bolivia, Paraguay, and Venezuela mention only the package insert for patients.

### Access to Information

Concerning the definition of an insert and its implications on the format for presenting the information, Table 2 shows that the definition in Central America [30, 31], Ecuador [38], Dominican Republic [54], Perú [53] and Paraguay [52] suggests this material is a supplement within the finished drug product, with a physical connotation. This might require an amendment for the implementation of electronic labels for inserts in the future since it is related to dissemination by digital media [16] and goes beyond current norms. Furthermore, CARICOM countries and Cuba do not specifically require a package insert, and the remaining countries in the region point out different guidelines for a physical insert in prescription drugs. ANVISA [24] requires the parallel implementation of information in a physical and an electronic format.

Meanwhile, the Internet has become one of the largest sources of health information for society and constitutes a path for its quick and dynamic distribution, reducing inequalities in access to information [68, 69]. When reviewing the presence of the approved prescribing information for drugs by the National Regulatory Authorities in their websites, it was observed that only Argentina, Brazil, Chile, Cuba, El Salvador and Peru present it. Thus, the rest of the countries need to publish this information on their websites to improve transparency and access to information.

According to its regulation, Brazil requires a timely update of the ANVISA site [24] and requires pharmaceutical companies to upload it in their domains. In Costa Rica, the information about reference products for therapeutic bio-equivalency was available at the time of this inquiry. The information posted on the DIGEMID (Perú) site is addressed to health professionals. The ANMAT, ANVISA and ISP sites present information for both patients and health professionals. However, not all countries show the information concerning their last update, so traceability is not feasible, and the rest of the countries offer no prescribing information publicly. This constitutes a great challenge for improving transparency as a pillar of Good Regulatory Practices [70]. In this regard, regulations from ANVISA [24], ANMAT, and DIGEMID establish that the National Regulatory Authority will make public any information approved through their websites.

Within the specific aspects of regulations in this region, the inclusive nature of the Brazilian regulation [24], stands out. It includes various characteristics and formats for populations with special needs and mandates to post the information on the websites of the pharmaceutical companies. These measures aim to address the difficulties of patients with visual problems to improve their adherence to treatments [71]. In some Health Authorities considered as regional references, such as Argentina, Chile, Colombia, Cuba, and Mexico, there is an evident need to improve their inclusive regulatory information for people with special needs. These countries do not incorporate the inclusive character as Brazil does in its existing regulations. Some shortcomings were identified in the structure and definition of the regulatory processes for managing the prescription information in Mexico, an important regional reference country.

Even when the regulation describes that we must comply with an insert for patients and another for health professionals, COFEPRIS has accepted the same content for both. However, when there is a specific insert for patients with a more simple language, the authority has accepted it.

### Regulatory Framework

In regulations, processes such as “*reliance*” or, in Spanish, “*el uso de decisiones reglamentarias de otras jurisdicciones*” was observed as the action by which a regulatory authority in one jurisdiction takes into account and gives significant importance to assessments made by another regulatory authority or reliable institution, or any other authorized information, to make its own decision [70]. Chile [26, 27] requires instructions for the prescribing information and for patients using EMA guidelines as a reference for both kinds of “information leaflets.” Argentina, Panama, and Peru require adapting the prescribing information according to what a reference regulatory authority approves as per the regulation [18, 20, 49, 53]. In the case of Argentina, something relevant to consider as a disadvantage is that changes cannot be implemented as soon as they are included in the Core Data Sheet, since it is necessary to wait for some reference authority to approve the change before submitting the update to ANMAT. The rest of the countries have no specific guidelines in this regard. However, although it might not be included in the regulation, it is possible that the adoption of the contents of the prescribing information approved by the reference regulatory authorities is required during the assessment.

Concerning changes related to the efficacy of prescription drugs, all Latin American and Caribbean countries require approval. As for Central American countries [30, 31] this kind of changes are managed through a headline in the Central American Technical Regulation (RTCA) Nº 11.03.59:11 entitled: Amendment A4 for Changes in the Monograph and Insert or A20 for the Extension of Therapeutic Indications. Meanwhile, Panama [49] does not follow this RTCA yet and instead follows Order 95, dated May 14, 2019, which applies a change in the monograph or insert or an update for the product’s efficacy. CARICOM countries lack regulations on this topic as well as concerning safety updates.

In Guyana and Bolivia, safety updates can be presented as notifications. As for Brazil, it all depends on the type of change taking place. In countries [30, 31] following the Central American Technical Regulation (RTCA) Nº11.03.59:11, safety updates take the form of an amendment established in B4 to change the product’s safety information. In Panama [49], these kind of changes require approval when submitted as a monograph or insert amendment but might be introduced as a notification of safety information change when generated in the sections of adverse reactions, contraindications, and warnings. The rest of the countries require approval.

In line with the need to define the contents of information on drugs, in Europe, a form named *Quality Review of Documents product information template* (QRD) was developed for the standardization of the structure and the text for pharmaceutical products information [72]. In the region of interest, Argentina and Brazil are the only countries with a standardized and mandatory structure for the presentation of drug information [18, 20, 24]. The rest of the countries present recommendations and lists of minimal sections in the prescribing information, with no structure or defined guidelines.

Thus, to adapt to changes in the field of innovative prescription medications and therapies in a context with various regulatory models [73], regulatory authorities will need to adopt an increased efficiency and address the challenges posed by the policies of the industry, which most likely will favor improved access to the products and therefore quicker access to the product information. Electronic labeling [16] constitutes an alternative that offers opportunities such as improved legibility and the ability to search for information on drugs, faster updating, and adaptation to other innovative technologies to be more customizable. Patients should benefit from all the potential of innovative technologies, and this solution makes highly relevant information available for the general public, a key element of Good Regulatory Practices [74].

## Conclusions

There is an evident need for harmonizing processes and standardizing the contents and the posting in official websites of the prescribing information for drugs in Latin America and the Caribbean. Also, we suggest adapting regulations to match the new technological developments, such as electronic labeling in the near future and seeking regional convergence options to build up in this regard. It may be useful to highlight the benefits of an agreed structure including improved opportunities for content re-use & automation; consistency in section names and numbers to ensure that healthcare professionals can quickly find key information.

Likewise, there is no consensus concerning whether the prescribing information for drugs is intended for health professionals or patients. Each country holds its own stand, which might limit access and make understanding difficult, such as different health literacy levels in these societies.

This paper also showed the diversity in the terms and guidelines included in inserts or leaflets for patients and health professionals for prescription drugs. Because of the lack of literature in this regard, we suggest, as next steps, assessing the efficacy of this kind of information for these audiences in the region.

No data on approval timelines for variations related to efficacy and safety were included because none were identified in the reviewed regulations. Additionally, the search for information on the regulatory authorities’ websites included in this study is not easily accessible. Also, the last update date is not present in most cases, so confirming whether the product information and package inserts posted were the latest authorized versions was not possible.

Despite the limitations, this is the first analysis of this type that has been conducted. We want to promote further development of this subject so that there is enough data to allow a contextual analysis since post-registration changes or safety and efficacy variations for a product might impact the prescribing information updates in real-time, access to this information is highly relevant. Providing complete, exact, and updated information for both patients and health professionals is of outmost importance. Every proposed solution shall consider both kinds of documents and aim at an improved system for information.
